# Monitoring side-effects of antipsychotics using the glasgow antipsychotic side-effect scale

**DOI:** 10.1192/bjo.2021.315

**Published:** 2021-06-18

**Authors:** James Sterritt

**Affiliations:** Dorset Healthcare University NHS Foundation Trust

## Abstract

**Aims:**

Antipsychotic drugs frequently produce side-effects which represent common reasons for noncompliance. National guidelines, published by the National Institute of Care and Health Excellence, the Royal College of Psychiatrists, and the Maudsley Prescribing Guidelines in Psychiatry, stipulate that patients prescribed antipsychotic drugs should be reviewed for side-effects on a weekly basis. This completed audit cycle, conducted on a mixed acute general adult psychiatric ward, examined whether patients were being assessed for side-effects of antipsychotic drugs using a standardised, self-reporting scale – the Glasgow Antipsychotic Side-effect Scale (GASS) – as per national guidelines. As identification of side-effects is important in tailoring treatment to improve compliance, auditing monitoring practice was important in realising these outcomes.

**Method:**

Retrospectively, 26 inpatients were identified over a two-month period who were prescribed antipsychotic drugs. Their notes were reviewed for documented weekly GASS scores for the duration of antipsychotic treatment. Initial data demonstrated 0% compliance with guidelines, as no patients completed a weekly GASS. The intervention to improve compliance was a training session for ward staff on implementing the GASS. Data were subsequently collected prospectively over three weeks for 15 patients.

**Result:**

Seven patients completed the GASS weekly over three weeks, representing 47% compliance. Two patients (13%) completed two forms, three (20%) completed one form, and three (20%) completed no forms. There was a positive correlation between being offered the GASS and completing it – only one patient declined to complete it and was not offered it during the third week. Of the remaining 14 patients, if the GASS was offered there was 100% rate of completion. Staff did not offer the GASS to every patient each week, which accounted for most cases of non-completion. Some patients with pre-existing symptoms of physical illnesses included these on the GASS, which complicated interpretation. Future interventions could include further staff education, and involving a ward pharmacist to review results during medication reviews to optimise treatment compliance, as no medication changes resulted directly from patients completing the GASS.

**Conclusion:**

Compliance with completing the GASS weekly improved following staff education, identifying the main factor affecting compliance as staff not offering the GASS to patients. Patients generally engaged well with side-effect monitoring, as most completed the GASS when offered. Further staff education may produce even greater compliance with guidelines, and involving pharmacy staff to review GASS scores and inform medication choices may lead to use of the GASS resulting in more tolerable and effective treatment plans.

